# Anchored and Propagated Updating Within Pseudoscientific Belief Systems

**DOI:** 10.1111/nyas.70229

**Published:** 2026-03-03

**Authors:** Josue García‐Arch, Marc Ballestero‐Arnau, Itxaso Barbería, Javier Rodríguez‐Ferreiro

**Affiliations:** ^1^ Departament De Cognició, Desenvolupament i Psicologia De l'Educació, Secció Processos Cognitius, Institut de Neurociències (UBneuro) Universitat De Barcelona (UB) Barcelona Spain; ^2^ Grup De Recerca en Cognició i Llenguatge (GRECIL), Departament de Cognició, Desenvolupament i Psicologia De l'Educació, Secció Processos Cognitius, Institut de Neurociències (UBneuro) Universitat De Barcelona (UB) Barcelona Spain

**Keywords:** belief updating, misinformation, pseudoscience, social learning, unwarranted beliefs

## Abstract

Pseudoscientific beliefs exert a profound influence on health behaviors, political decisions, and public trust in science, yet research has primarily identified correlates of pseudoscience acceptance rather than the mechanisms by which such beliefs form and update. In this study, we leveraged computational modeling to investigate how normative social feedback shapes pseudoscientific belief revision. A total of 300 US nationally representative participants conducted a learning task where they rated a set of 20 validated pseudoscientific statements while receiving trial‐by‐trial feedback. Behaviorally, participants showed systematic reductions in prediction errors across trials, consistent with iterative belief updating. Computational model comparison using hierarchical Bayesian inference revealed that learning was best captured by an anchored propagation model, in which prediction errors spread across correlated beliefs but were stabilized by an anchoring parameter reflecting initial convictions. Exploratory analyses further showed that belief updating depended on the alignment between prior expectations and normative feedback, amplifying congruent information and dampening incongruent inputs. These findings provide the first mechanistic account of how pseudoscientific beliefs are simultaneously receptive to new information and resistant to change, offering an integrative framework with implications for research in belief updating, social cognition, and interventions to reduce misinformation.

## Graphical abstract

Belief revision in pseudoscience reflects structured interactions between prior convictions and social information. Using a U.S. representative sample and computational modeling, we found that the effects of normative feedback spread across correlated beliefs but remain constrained by anchoring to initial views. This anchored propagation mechanism explains how misinformation endures and suggests that targeted interventions can leverage the network structure of beliefs to promote broader conceptual change.

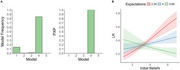

## 1 Introduction

Beliefs in pseudoscientific claims have profound and widespread influence in critical life choices, including dangerous health practices, political decisions, and societal attitudes toward science. The infiltration and proliferation capabilities of pseudoscience pose a significant barrier to public understanding of evidence‐based knowledge and can undermine decision‐making processes at both the individual and societal levels [1, 2, 3, 4, 5]. Indeed, the endorsement of pseudoscientific beliefs and practices such as those related to vaccine hesitancy, astrology, pseudo‐psychology, or alternative medicine have been linked to tangible societal harms [2, 3, 4, 6, 7, 8, 9, 10]. Therefore, elucidating the cognitive mechanisms by which individuals form and adjust pseudoscientific beliefs represents a pressing scientific and societal need.

Previous research in this field has primarily focused on identifying demographic, psychological, and social factors associated with susceptibility to pseudoscientific beliefs, including cognitive biases [11, 12, 13, 14, 15, 16, 17], educational background [18, 19], personality traits [18, 20], cognitive styles [18, 21, 22, 23], personal and political attitudes [24, 25, 26], and exposure to pseudoscientific information [18]. This research has made a significant and successful effort to identify who is more likely to endorse these unfounded beliefs. However, our knowledge on the cognitive mechanisms underlying the formation and revision of pseudoscientific beliefs is scarce. The delineation of such mechanisms is crucial, as it might offer new pathways to refining ongoing efforts to combat the widespread influence of pseudoscience by tailoring existing initiatives to the underlying cognitive processes of their recipients [20, 27, 28, 29].

An intriguing and widely observed phenomenon in the study of pseudoscientific beliefs is their tendency to converge into coherent sets of inter‐relationships. Remarkably, individuals who endorse one pseudoscientific claim often show a greater likelihood of endorsing multiple related pseudoscientific beliefs. This structured correlation across seemingly distinct pseudoscientific statements is robust enough to have led researchers to develop validated psychometric scales specifically aimed to measure these inter‐related beliefs [15, 18, 21, 30, 31]. While this intercorrelation has been largely absorbed by researchers as a natural and even necessary property for its measurement, it also presents an unexplored opportunity for understanding whether information received from the environment about a specific pseudoscientific claim might propagate to other correlated beliefs. From a cognitive and computational perspective, such propagation is plausible because human belief systems are based on correlated elements, and prediction errors (PEs) associated with one of them can lead to simultaneous updating in related elements [32, 33, 34, 35, 36]. In practice, this means that encountering new, discrepant information about a particular pseudoscientific claim could trigger a cascade of belief revision. Moreover, from a measurement perspective, the very logic underpinning the construction of psychometric instruments demands that items grouped within a scale exhibit meaningful intercorrelation. This principle ensures that the measurement is capturing a coherent latent construct. In this sense, the propagation of informational effects across related beliefs is a plausible expectation rooted in the psychometric architecture of how psychological constructs are operationalized and assessed.

Here, we leveraged recent advances in computational modeling of multifaceted constructs [33, 34, 35, 36] to test the hypothesis that when individuals encounter environmental information about specific pseudoscientific claims, learning propagates through the broader belief system, resulting in a reduction of discrepancies between participants’ beliefs and reference feedback over the course of a learning task. Recent work has shown that computational models can successfully capture how people learn about multifaceted constructs such as the utilities associated with character traits [33], the personality and preferences of other individuals [34, 35, 36], or social roles [32]. At their core, these computational approaches formalize the process by which participants update their expectations for one element and propagate PEs to structurally related elements, using a similarity matrix derived from empirical correlations, combined with the estimation of a learning rate as a free parameter. However, this framework is sufficiently flexible to incorporate other sources of personal or social knowledge [33, 34, 37], including the influence of the individuals’ initial beliefs. By embedding participants’ baseline endorsement of pseudoscientific claims in a model and parametrizing its modulatory effect, we could open the door to quantitatively assess incremental learning effects based on the propagation of PEs, offering a mechanistic account of belief updating within the architecture of pseudoscientific beliefs.

In this research, we asked participants to undergo a learning task where they learned from normative population‐level social feedback about pseudoscientific statements. We hypothesized that participants would adjust their endorsement of individual pseudoscientific statements in response to such feedback, thereby effectively reducing the distance between their ratings and those coming from a reference group through the course of the task. Moreover, we tested several computational models aimed to capture the mechanisms involved in iterative pseudoscientific belief‐updating.

## 2 Methods

### 2.1 Participants

Prior studies [33, 35] indicate that samples of 40–90 participants are sufficient for testing models of similar complexity. However, we increased the sample size to 300, which is the minimum required for the recruitment of a representative sample (sex, age, and ethnicity among US residents) in Prolific (153 females, *M* age = 46.14, *SD* age = 15.63). All participants provided informed consent after reading a detailed information sheet. The protocol was approved by the University of Barcelona Bioethics Committee (IRB00003099). This study was preregistered on the Open Science Framework (https://aspredicted.org/v94g‐r6xs.pdf). Data were analyzed using MATLAB (version 2024b).

### 2.2 Procedure

**FIGURE 1:**
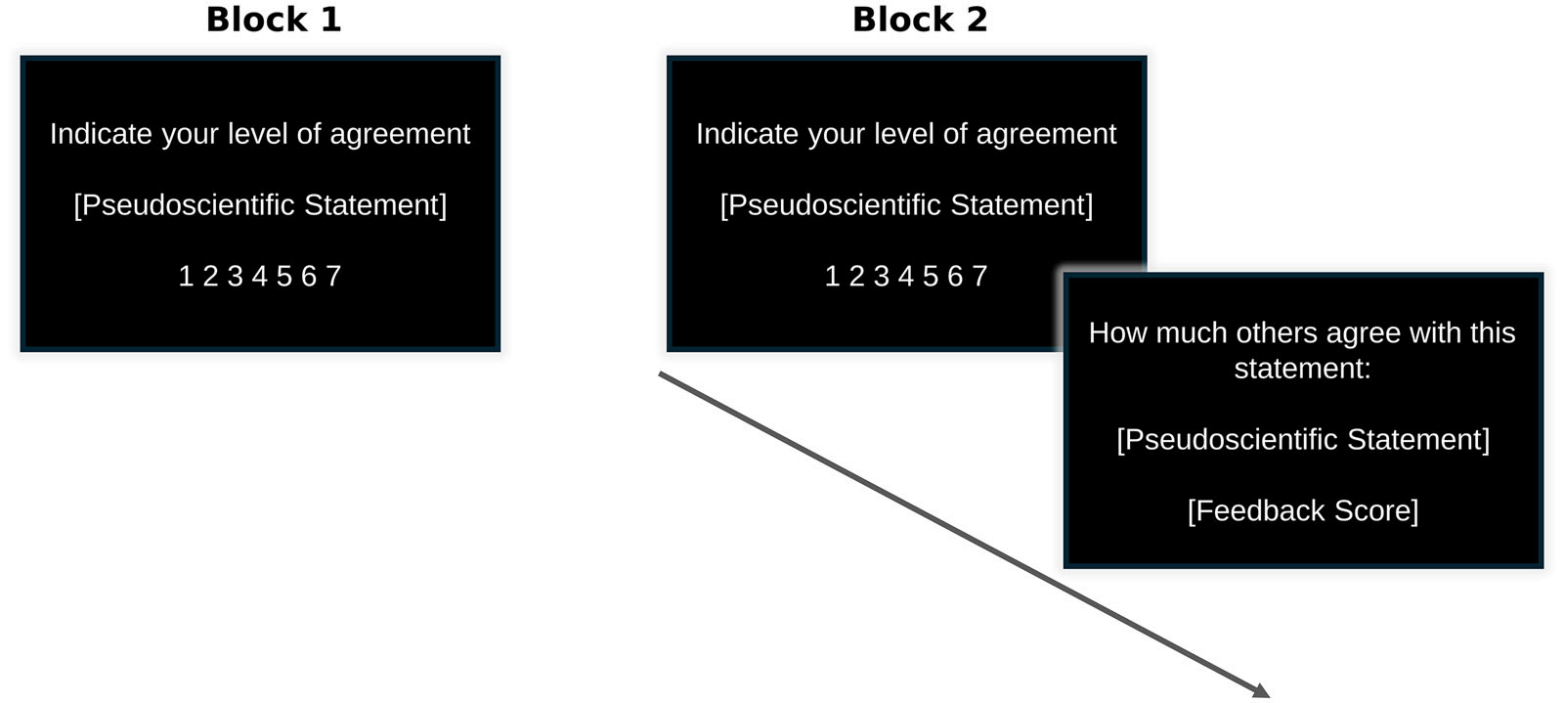
Experimental task. In Block 1, participants rated their agreement with pseudoscientific statements (e.g., “The manipulation of energies bringing hands close to the patient (for example, Reiki) can cure physical and psychological maladies”) on a 1–7 scale. After a brief distractor task, the same statements were presented again in Block 2. Immediately after each rating, participants received normative feedback indicating how strongly a nationally representative US sample agreed with that same statement. For example, after rating the statement above (“The manipulation of energies bringing hands close to the patient can cure physical and psychological maladies”), participants might see a screen displaying: “How much others agree with this statement”: “The manipulation of energies bringing hands close to the patient (for example, Reiki) can cure physical and psychological maladies,” with a score below (e.g., 3.71), representing the average agreement score. Feedback always mirrored the exact statement presented and reported the numerical consensus score to two decimal places.

Participants conducted a social learning task inspired by prior studies [32, 33, 35]. The task consisted of two blocks. In the first block, participants provided their beliefs about 20 pseudoscientific statements extracted from a validated instrument [15, 30]. In the second block, they provided again the same ratings, while receiving average normative feedback from a nationally representative sample (*n* = 510, stratified by age, sex, and ethnicity) from the United States [30] (Figure 1). Statements were randomized across participants. At the beginning of each trial, participants encountered the prompt “Indicate your level of agreement” accompanied by one pseudoscientific statement (e.g., “The manipulation of energies bringing hands close to the patient (for example, Reiki) can cure physical and psychological maladies”). Below this prompt, a 7‐point Likert scale was provided for participants to rate how much they agreed with the statement, with 1 indicating “Strongly Disagree” and 7 meaning “Strongly Agree” (self‐paced). After the first block, participants performed a distractor task consisting of simple mathematical operations. Next, during the second block, participants were again provided with their agreement with the same pseudoscientific statements while receiving trial‐by‐trial normative feedback. That is, directly after their estimations, participants received feedback consisting of the average estimates of a reference group. Participants were informed that this feedback came from a US nationally representative sample, and that the scores represented the average evaluations participants made in a prior study [30]. The feedback appeared on the screen in the form “How much others agree with this statement”: followed by the same statement participants just evaluated, and a score ranging from 1 to 7, with a maximum of two decimals. This sequence was repeated for the 20 statements included in the pseudoscience beliefs scale. Importantly, participants were not instructed to learn during the task. Task instructions are reported in Note S1.

### 2.3 Stimuli

Stimuli consisted of 20 pseudoscientific statements extracted from a validated questionnaire (e.g., “The manipulation of energies bringing hands close to the patient (for example, Reiki) can cure physical and psychological maladies.”) [15, 30]

### 2.4 Computational Models

We tested five computational models to explore which model best described participants’ learning strategies. Our models were inspired by recent research in learning multidimensional constructs such as the utilities of character traits, personalities and preferences of other individuals, and social roles [35, 36, 38, 39]. This research indicates that when learning about multifaceted constructs, participants use fine‐grained, interelement relationships to spread PEs and promote learning. Here, this learning mechanism (so‐called, fine granularity learning) entails the adjustment of expectations for upcoming feedback on pseudoscientific statements based on the discrepancy between the participant's estimation of a given statement and the feedback received (i.e., PE) via a similarity matrix (Figure S1). Further, this update is modulated by the learning rate, which is estimated as a free parameter for each participant. The similarity matrix included in our models (SIM), together with the feedback ratings, were computed from a large separate sample (*n* = 510) from the same population [30]. Four of our five computational models were operationalized as hybrid reinforcement learning models, including, but not limited to, a fine granularity learning mechanism. The remaining model consisted of a simple regression that assumes no learning, that is, participants’ final pseudoscience beliefs are only a function of their initial beliefs. Models 2−5 make use of the PE to update the prediction (P) for the following trials. The PE for all models is the feedback (F) on a certain trial (t) minus the prediction (P) on that trial (see below for details). Initial feedback expectations (E) were defined by treating the first statement's expectation as a free parameter, and subsequent expectations were adjusted via linear regressions based on the interitem similarity matrix [38]. Extensive details of the fitted models are reported below.

#### 2.4.1 Model 1: No Learning

Model 1 assumes that participants do not learn. This model performs like a standard linear regression where β0 represents the intercept, β1 the slope, and IB represents participants initial beliefs.

P=β0+β1·IB

#### 2.4.2 Model 2: Fine Granularity

Model 2 employs fine‐grained granularity, allowing a PE on one pseudoscientific statement to propagate to other statements in the task, in proportion to their empirical interitem correlations. That is, on a trial‐by‐trial basis, Model 2 updates the estimates of upcoming statements based on the current PE. Moreover, it weighs the spread of the PE to upcoming statements by means of the learning rate. This interconnected updating aligns with prior theoretical and computational research [32, 33, 34, 35]. Here, E(t) stands for the initial feedback expectation for the current trial t, α for the learning rate, and SIM for the similarity matrix.

$$P(t+1)=E(t)+\sum_{i=2}^{t-1}\alpha \cdot \mathrm{PE}(i)\cdot \mathrm{SIM}(i,t+1)$$

#### 2.4.3 Model 3: Fine Granularity, Asymmetric Learning (Two Learning Rates)

Model 3 extends Model 2 by permitting differential sensitivities to positive and negative PEs. On each trial, the discrepancy between received feedback and expectation is computed for the current statement. Depending on its sign, this error is scaled by either a positive learning rate or a negative learning rate and then propagated to other statements in proportion to their empirical interitem correlations (SIM). This structure allows us to account for differential upward versus downward belief revision.

$$P(t+1)=E(t)+\sum_{i=2}^{t-1}\alpha \left\{+,-\right\}\cdot \mathrm{PE}(i)\cdot \mathrm{SIM}(i,t+1)$$

#### 2.4.4 Model 4: Anchored Fine Granularity

Model 4 extends Model 2 by integrating each participant's baseline beliefs (IB) as an anchoring reference on every trial while accounting for incremental learning effects that balance new information against initial beliefs. Here, the final outcome is a weighted average of the initial belief and the propagation‐based update, governed by a participant‐specific parameter *γ* (bounded 0–1). Higher *γ* values favor stability around initial beliefs, preventing shifts when feedback diverges, whereas lower *γ* values allow greater incorporation of external information.

$$P^{m}(t+1)=E(t)+\sum_{i=2}^{t-1}\alpha \cdot \mathrm{PE}(i)\cdot \mathrm{SIM}(i,t+1)$$

$$P\left(t\right)=\mathrm{IB}\left(t\right)\cdot \gamma +\left(1-\gamma \right)\cdot P^{m}\left(t\right)$$

#### 2.4.5 Model 5: Anchored Fine Granularity (Two Learning Rates)

Model 5 combines Model 4 with the dual learning rates from Model 3.

$$P^{m}(t+1)=E(t)+\sum_{i=2}^{t-1}\alpha \left\{+,-\right\}\cdot \mathrm{PE}(i)\cdot \mathrm{SIM}(i,t+1)$$

$$P\left(t\right)=\mathrm{IB}\left(t\right)\cdot \gamma +\left(1-\gamma \right)\cdot P^{m}\left(t\right)$$

### 2.5 Model Fit and Comparison

Model fitting and comparison were conducted using the hierarchical Bayesian inference (HBI) framework, which provides simultaneous estimation of both individual‐ and group‐level parameters, while flexibly assigning model identity as a probabilistic latent variable. This approach moves beyond conventional fixed‐effect methods by allowing each participant's data to contribute to the estimation of population distributions and to the determination of which candidate model most plausibly accounts for their behavior [40]. Specifically, model assignment is treated as a multinomial variable for each subject, reflecting the relative evidence in favor of each computational hypothesis and enhancing robustness to outliers. HBI facilitates direct comparison of competing models through metrics such as the protected exceedance probability (PXP), which represents the probability that a given model is superior, after accounting for chance [40, 41]. All analyses were implemented in MATLAB (v2024b) using the Computational and Behavioral Modeling toolbox (https://payampiray.github.io/cbm), and parameter estimation employed wide Gaussian priors [40]. To assess the robustness of our computational models, we conducted parameter recovery analyses by simulating 200 datasets with randomly drawn parameters and post‐simulation added noise. The correlations for parameters within our best‐performing model (Model 4) were robust: (learning rate: *r* = 0.826; gamma: *r* = 0.963, E: *r* = 0.901; correlations between simulated and recovered parameters for all models are presented in Figure S2). We further simulated 200 datasets per model to construct a confusion matrix and assess model recovery, that is, each model's ability to be correctly recognized from its own simulated data. Using PXP as the criterion, we found perfect separation: the data‐generating model was always identified as the best fit (identity matrix), demonstrating robust model distinguishability.

## 3 Results

We started by evaluating whether participants exhibited learning during the task. To that end, we first tested for a systematic reduction in PEs over time, a foundational indicator of iterative belief updating [33, 35]. To flexibly capture potential nonlinear trajectories in PE as a function of trial number, we employed generalized additive models (GAMs), which allow for data‐driven estimation of smooth trends without imposing restrictive parametric assumptions. Specifically, we constructed a model with PEs as a dependent variable and time as an independent variable (smooth term); participants’ IDs were introduced as a random effect. Further, we parametrically controlled for participants’ initial beliefs (*M* = 4.06, *SD* = 0.97, possible range 1−7). Approximately 14% of participants scored above 5 on average, and 15% scored below 3, indicating that the sample captured a reasonable and heterogeneous spectrum of endorsement. Consistent with the hypothesis that exposure to feedback would drive iterative belief updating, the GAM revealed a significant smooth term for time (*X* ^2^(1)  =  10.47, *p*  =  0.0012), reflecting a decrease in PEs throughout the task (Figure S3).

**FIGURE 2:**
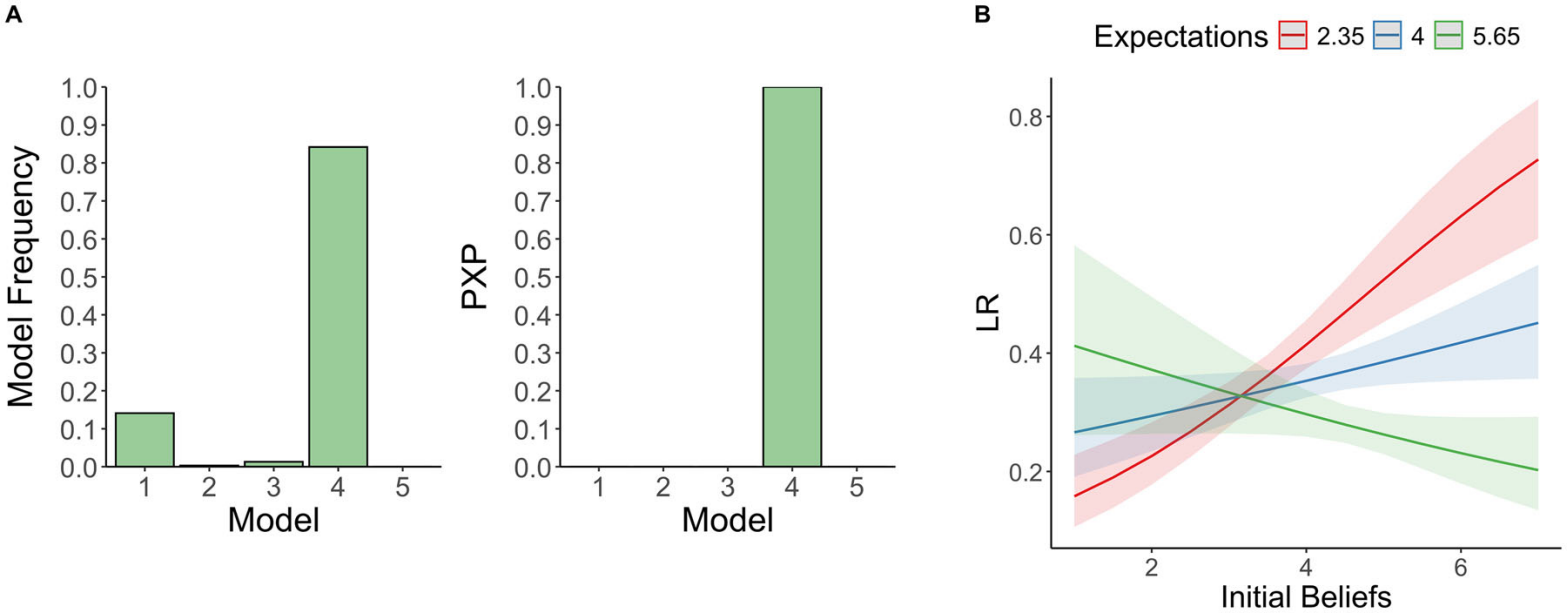
Model comparison and moderation of learning rates by initial beliefs and expectations. (A) Results from the hierarchical Bayesian inference (HBI) model comparison, showing model frequency (left) and protected exceedance probability (PXP) (right) across the five candidate models. (B) Moderation analysis of learning rates (LRs) as a function of participants’ initial beliefs and their starting expectations. Lines depict fitted values from the regression model, and shaded areas represent 95% confidence intervals. For visualization, the continuous expectations variable was split into representative values (mean ± 1 SD) following the standard convention when plotting interactions between quantitative predictors.

Next, we conducted an HBI analysis to determine which computational model best accounted for participants’ belief‐updating behavior. Results identified Model 4, the Anchored Fine‐Granularity Model as the best fitting model based on model frequency (Model 1: 0.142, Model 2: 0.004, Model 3: 0.013, Model 4: 0.841, Model 5: 0), model parameters: learning rate (*M* = 0.297, *SD* = 0.222), gamma (*M* = 0.727, *SD* = 0.232), Expectations (*M* = 4.009, *SD* = 1.652). The PXP, which quantifies the likelihood that a given model provides a better fit than any competitor in the model space after correcting for chance, confirmed this result, providing strong evidence of the superiority of the winning model (Model 1: 0, Model 2: 0, Model 3: 0, Model 4: 1, Model 5: 0.0, Figure 2A). Model 4 integrates each participant's initial beliefs as a reference point, allowing belief updates to propagate through the interconnected pseudoscientific beliefs while anchoring changes to individuals’ initial beliefs. Next, we evaluated the capacity of the winning model to recapitulate participants’ behavioral trajectories through group‐level posterior predictive checks. Parameters were sampled from the posterior distribution, and trial‐by‐trial estimation trajectories were simulated for each dataset. The 95% confidence intervals of these model‐generated trajectories were then compared against the empirical mean trajectories and the model's posterior‐predictive means to assess correspondence. The empirical data fell within the model's confidence intervals, and the posterior‐predictive mean closely tracked the observed time course (Figure S4).

Finally, we conducted an exploratory analysis to investigate the relationship between individuals’ initial beliefs and the three computational parameters derived from Model 4. For each computational parameter, we fitted a robust beta regression in which the parameter served as the dependent variable, and participants’ initial beliefs were entered as the primary predictor, adjusting for the remaining two computational parameters as covariates. Robust beta regression was chosen for its suitability in modeling continuous bounded outcomes, as well as its resilience to deviations from model assumptions. The robust estimation procedure utilizes a data‐driven tuning constant to optimally balance statistical efficiency and robustness, thereby minimizing the undue influence of departure from classical model assumptions on coefficient estimates and inference [42]. Results indicated that initial beliefs were positively associated with the learning rate (*β* = 0.137, *SE* = 0.066, *z* = 2.099, *p* = 0.035), such that higher baseline endorsement of pseudoscientific statements was linked to a greater sensitivity to feedback during the task. Starting expectations were negatively associated with the learning rate (*β* = −0.171, *SE* = 0.066, *z* = −2.582, *p* = 0.009), whereas gamma showed no significant association (*β* = 0.047, *SE* = 0.056, *z* = 0.859, *p* = 0.390). Results also indicated that initial beliefs were positively associated with starting expectations (*β* = 0.943, *SE* = 0.079, *z* = 11.943, *p* < 0.001) and not significantly associated with the anchoring parameter (*β* = 0.059, *SE* = 0.070, *z* = 0.847, *p* = 0.397).

Further, we explored whether the influence of participants’ initial beliefs on their learning rate varied depending on the congruence between expectations and social feedback. Note that “congruent” represents those instances where the direction of the feedback moved participants’ perceived social consensus in line with their baseline beliefs, and “incongruent” represents those where feedback moved it in the opposite direction. To that end, we constructed an additional robust beta regression including a belief × starting expectation interaction term, while controlling for gamma. Results revealed a positive but nonsignificant main effect of initial beliefs (*β* = 0.120, *SE* = 0.063, *z* = 1.895, *p* = 0.058). Starting expectations exerted a significant negative main effect (*β* = −0.182, *SE* = 0.062, *z* = −2.899, *p* = 0.003). Crucially, the belief × starting expectation interaction was significant and negative (*β* = −0.375, *SE* = 0.059, *z* = −6.328, *p* < 0.001), indicating that the positive association between beliefs and learning rate observed at lower starting expectations reversed at higher starting expectations. Probing the interaction (Figure 2B) revealed that when expectations about others’ agreement were below the actual social consensus (e.g., E ≈ 2.36< F = 3.711), higher initial beliefs predicted stronger learning rates (red line). When expectations were above consensus (e.g., E ≈ 5.66), higher initial beliefs predicted weaker learning rates (green line). Thus, learning was amplified when feedback moved perceived consensus in the same direction as participants’ beliefs (consensus‐congruent) but dampened when it moved in the opposite direction (consensus‐incongruent).

## 4 Discussion

Our findings provide converging behavioral and computational evidence that updating pseudoscientific beliefs is not a simple, isolated process but instead reflects iterative adjustments. Across a large, demographically diverse sample, we found that participants systematically reduced their PEs over the course of a learning task, and computational modeling revealed that this reduction was best captured by an anchored granularity mechanism: feedback effects propagated to related beliefs in proportion to their empirical associations, while updates were regulated by a participant‐specific anchoring to initial beliefs. This mechanism aligns with contemporary computational views of similarity‐based belief updating as partially self‐stabilizing yet receptive to new information [32, 33, 34, 35].

From a measurement perspective, these findings highlight a critical implication, namely, the psychometric structure that allows researchers to capture pseudoscientific beliefs as a coherent construct [15, 18, 21, 30] also provides a pathway for environmental information to influence multiple beliefs simultaneously. Interestingly, this finding could imply that changes introduced at the level of specific statements may generalize beyond those items, or even constructs, to other unwarranted beliefs that share overlapping variance with pseudoscience acceptance, such as paranormal claims, illusory health beliefs, or conspiracy theories [31, 43, 44]. In principle, this structural property represents both a risk and an opportunity, since it can accelerate the spread of misinformation if the initial input is false, but it could also amplify corrective information if carefully targeted.

Our exploratory moderation analysis revealed a continuous interaction between initial beliefs and social expectations. As initial beliefs increased, learning rates rose when baseline expectations were low, remained relatively stable when expectations were moderate, and declined when expectations were high. This pattern suggests that strong prior beliefs do not preclude belief revision per se, but rather modulate the magnitude of learning depending on how feedback aligns with prior expectations. We modeled this interaction continuously both to provide rich coverage of the effect and because the pseudoscientific belief scale used in this work was validated as a continuous measure, and currently, there is no established criterion for partitioning individuals into discrete belief categories (e.g., strong vs. weak believers). This pattern indicates that the capacity to update is jointly shaped by individuals’ starting expectations, with certain configurations of belief strength and expectations fostering receptivity, while others are associated with reduced responsiveness to feedback. This pattern is consistent with a framework of selective integration of social‐consensus information, in which feedback that is consensus‐congruent is more readily incorporated, whereas consensus‐incongruent feedback is integrated to a lesser degree [45, 46]. Future work should manipulate the source of consensus feedback, for example, comparing identity‐congruent and population‐level norms, in order to examine the conditions under which anchored and propagated updating is sensitive to the perceived origin of normative information.

While unwarranted beliefs are often conceptually linked to political ideology or cultural identity, many such conclusions derive from stimulus sets composed largely of highly politicized topics (e.g., vaccines, climate change). The present study employed a validated scale intended to measure pseudoscientific endorsement as a latent construct [15, 30] rather than to contrast politically charged and neutral subdomains, and thus cannot directly speak to whether certain topics elicit greater receptivity or resistance to consensus feedback. However, our findings raise several theoretically tractable possibilities for future work. If political salience primarily strengthens anchoring to prior beliefs, this would imply domain‐general updating but heterogeneous magnitudes of change. Alternatively, politicized beliefs may constitute distinct informational clusters with altered covariance structures [18], which could yield domain‐specific patterns of propagation. Designing stimulus sets with multiple items per topical category and preregistered domain‐level hypotheses will be necessary to determine whether anchored and propagated updating varies systematically as a function of ideological relevance, and to assess whether the similarity architecture of belief networks remains invariant across political identities.

Generally, our findings have practical implications for the design of science‐communication and public‐health interventions. Because belief revision seems to be anchored to prior convictions and generalize along interbelief similarities, corrective strategies may be more effective when they (1) account for the baseline endorsement to calibrate feedback strength, (2) target sets of correlated beliefs rather than isolated claims, and (3) prioritize “hub” statements with strong connectivity to maximize downstream impact [28]. Framing feedback as incremental, cumulative evidence rather than a single, categorical correction may further facilitate alignment with corrective information. More broadly, interventions that account for the similarity structure of pseudoscientific beliefs may produce larger and more durable shifts than one‐size‐fits‐all messaging.

A deeper theoretical question arises when considering the nature of these changes from the perspective of measurement theory. The interitem correlations in pseudoscience belief scales reflect the presence of a latent common factor, often interpreted as “susceptibility to pseudoscience” or a broader *epistemic style* favoring unfounded claims. In turn, our results show that updating one belief can influence others through this structural linkage, but whether this reduces the underlying latent factor is a more complex question. If the common factor truly reflects a stable cognitive disposition [2, 15, 23], for example, a preference for intuitive over analytical reasoning, or a generalized openness to unfounded claims, then adjusting item‐level beliefs may only produce transient changes unless the underlying disposition is also addressed. On the other hand, if the common factor is at least partly constructed from the aggregation of specific beliefs, then altering those beliefs could, in principle, shift the latent construct itself. Disentangling these possibilities is not merely a statistical challenge but a conceptual one, touching on the rationale of measurement principles: is susceptibility to pseudoscience a causal driver of specific endorsements, or an emergent property of them? (or both).

This distinction carries important practical implications. If pseudoscientific beliefs are rooted in a stable cognitive disposition, such as a general tendency toward causal reasoning or intuition [16, 17, 18, 21], efforts must directly target epistemic styles, critical thinking, and meta‐cognitive norms. Conversely, if the latent susceptibility factor is substantially shaped or sustained by the structural interdependence of its constituent beliefs, then shifting beliefs on specific statements might propagate through the network, yielding broader conceptual transformation via propagation.

Our findings suggest that latent epistemic disposition constrains change, yet the structural architecture of belief networks may allow targeted interventions to infiltrate throughout the system. This perspective aligns with educational studies showing considerable reductions in paranormal and pseudoscientific beliefs following critical‐thinking courses tailored to enhance reasoning skills and confront pseudoscientific content directly [28]. Likewise, research highlights substantial heterogeneity in pseudoscientific health beliefs and underscores the necessity of tailoring correction strategies to align with individuals’ underlying causal reasoning patterns [16]. Together, these findings support the notion that combining interventions aimed at core epistemic tendencies with strategic targeting of pivotal beliefs may offer the most effective route to durable belief change.

The anchoring parameter in our best‐fitting model can thus be viewed as a quantitative index of resistance to change, that is, a tendency to weight existing beliefs over incoming evidence. In a structurally interdependent belief system, such conservatism may prevent radical shifts in worldview but also slow the spread of corrective information. The balance between propagation (which allows new information to influence multiple‐related beliefs) and anchoring (which stabilizes beliefs against change) could, therefore, determine the trajectory of belief revision at both the individual and population level.

Finally, the methodological contribution of this work lies in demonstrating that computational models developed for learning about multifaceted constructs (e.g., character traits, traits’ utilities, and social roles) can be successfully applied to belief updating in the context of pseudoscientific information, yielding mechanistic parameters that can be related to individual differences. By grounding our similarity matrix in empirical data and testing models within a hierarchical Bayesian framework, we ensured that model selection was robust to individual variability and outliers. This approach opens opportunities for tracking belief change over time, simulating the potential impact of interventions, and identifying individuals or subgroups most likely to respond to targeted information strategies. A further consideration concerns how anchored and propagated updating operates in information environments where consensus signals are not always clear, trusted, or aligned with individuals’ expectations. Our task presented consensus feedback explicitly and repeatedly, enabling us to isolate the underlying learning mechanism once consensus information is treated as a relevant reference point. Our findings suggest that updates need not be delivered exhaustively nor uniformly across all propositions to produce wider conceptual change; when beliefs are structurally coupled, even sparse or domain‐localized signals can propagate through the network, shaping unobserved beliefs via their inferred similarity to those that are directly updated. However, in some domains, individuals may reject not only the substance of scientific conclusions but also the existence of consensus itself, as shown in work on genetically modified foods, where extreme opponents exhibit low objective knowledge yet high perceived understanding and frequently dispute the consensus position [47]. Extending the present framework to incorporate meta‐beliefs, including beliefs about what experts or the majority truly think, and beliefs about the reliability or motives of those sources, would allow future research to examine how changes in content beliefs and beliefs about the epistemic environment coevolve. Such an approach could help clarify to which extent resistance to corrective information reflects conservative anchoring of existing beliefs, uncertainty about the underlying consensus, and updating dynamics driven by the perceived trustworthiness and identity relevance of the source.

In sum, our findings underscore the value of viewing pseudoscientific beliefs, as well as related unwarranted beliefs, not as isolated misconceptions but as structurally interdependent statements whose revision emerges from the interplay between the propagation of new information and the stabilizing influence of prior commitments and social expectations. Recognizing and leveraging this interplay may be key to designing more effective, psychologically informed interventions to reduce the societal impact of pseudoscience and other forms of misinformation.

### 4.1 Limitations

Although our findings provide robust evidence for an anchored propagation mechanism in pseudoscientific belief updating, several considerations delimit the scope of the conclusions. First, our task was conducted in a controlled experimental setting using concise pseudoscientific statements and normative feedback. While this affords precision and internal validity, future work should examine whether the same computational principles extend to more naturalistic contexts, such as media exposure or interpersonal discussion. Although our sample was large and demographically stratified to reflect the US population, replication in other cultural settings will be important for assessing the generality of the proposed mechanism across societies. In our paradigm, the reference group instantiated by the feedback was a nationally representative sample of US adults. However, for some individuals with strong pseudoscientific commitments, day‐to‐day normative input may come from narrower, identity‐relevant communities that are more trusted than population‐level information. Future studies should, therefore, vary the source of the consensus feedback, for example, by contrasting ingroup‐based and general population norms, to test whether anchored and propagated updating depends on the perceived social identity of the reference group. Finally, the present design focused exclusively on pseudoscientific claims. Incorporating established scientific claims under analogous feedback conditions would help determine whether the dynamics of anchored and propagated updating are domain general or shaped by the perceived epistemic status of the belief itself.

## Author Contributions

J.G.‐A. led the formal analysis, visualization, and writing of the original manuscript. J.G.‐A., M.B.‐A., I.B., and J.R.‐F. jointly contributed to the conceptualization of the study. M.B.‐A. implemented the experimental task, managed data collection, and contributed to data curation. I.B. and J.R.‐F. provided supervision and secured funding. All authors contributed to the revision and editing of the manuscript and approved the final version.

## Conflicts of Interest

The authors declare no conflicts of interest.

## Supporting information




**Supplementary Material**: nyas70229‐sup‐0001‐SuppMat.docx

## Data Availability

Data and analysis code are available at the Open Science Framework (OSF). Link: https://osf.io/68wrt/?view_only=248a60100d824e7bbcc42c6fc3dd2bc7
