# Supplementary material for: Anchored and Propagated Updating Within Pseudoscientific Belief Systems
Source: Ann N Y Acad Sci. 2026 Mar 3;1557(1):e70229. doi: 10.1111/nyas.70229 (PMC12955757; doi:10.1111/nyas.70229)
Supplement: Supplementary file 1 — Supplementary Material: nyas70229‐sup‐0001‐SuppMat.docx [file NYAS-1557-0-s001.docx]

| 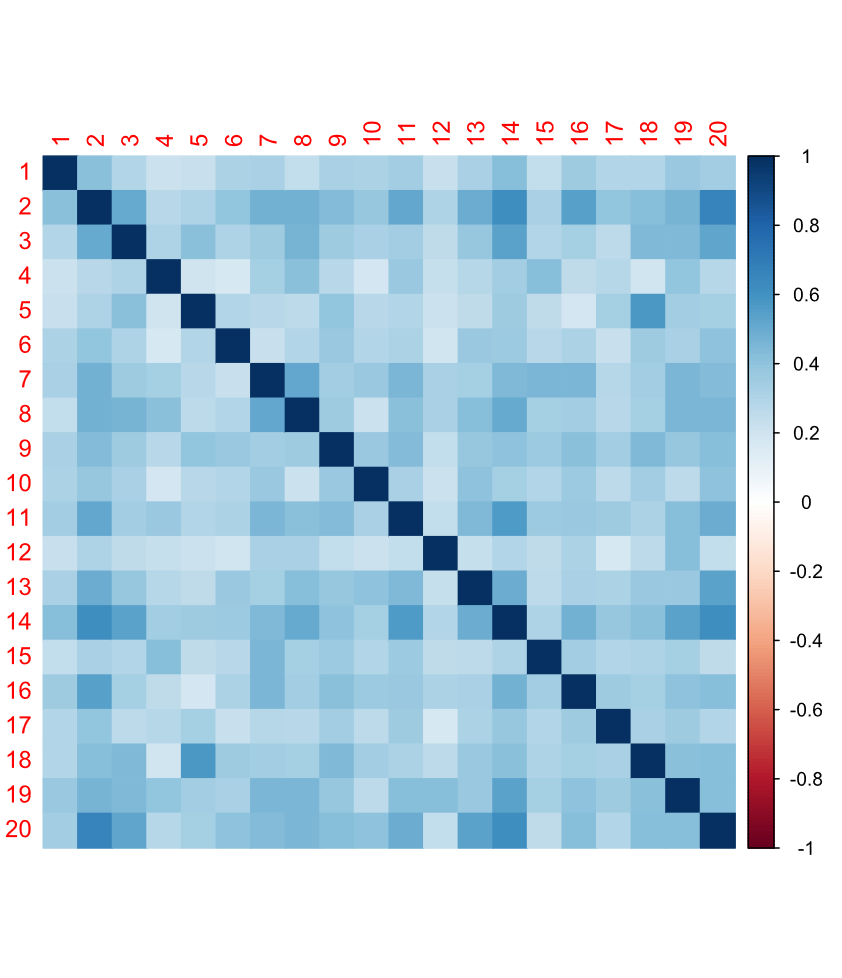 |
| --- |
| **Figure S1.** Similarity Matrix employed in computational models (SIM). This similarity matrix comes from a normative sample from the same population and was employed to validate the psychometric scale in a prior study (Torres et al., 2023) |

| **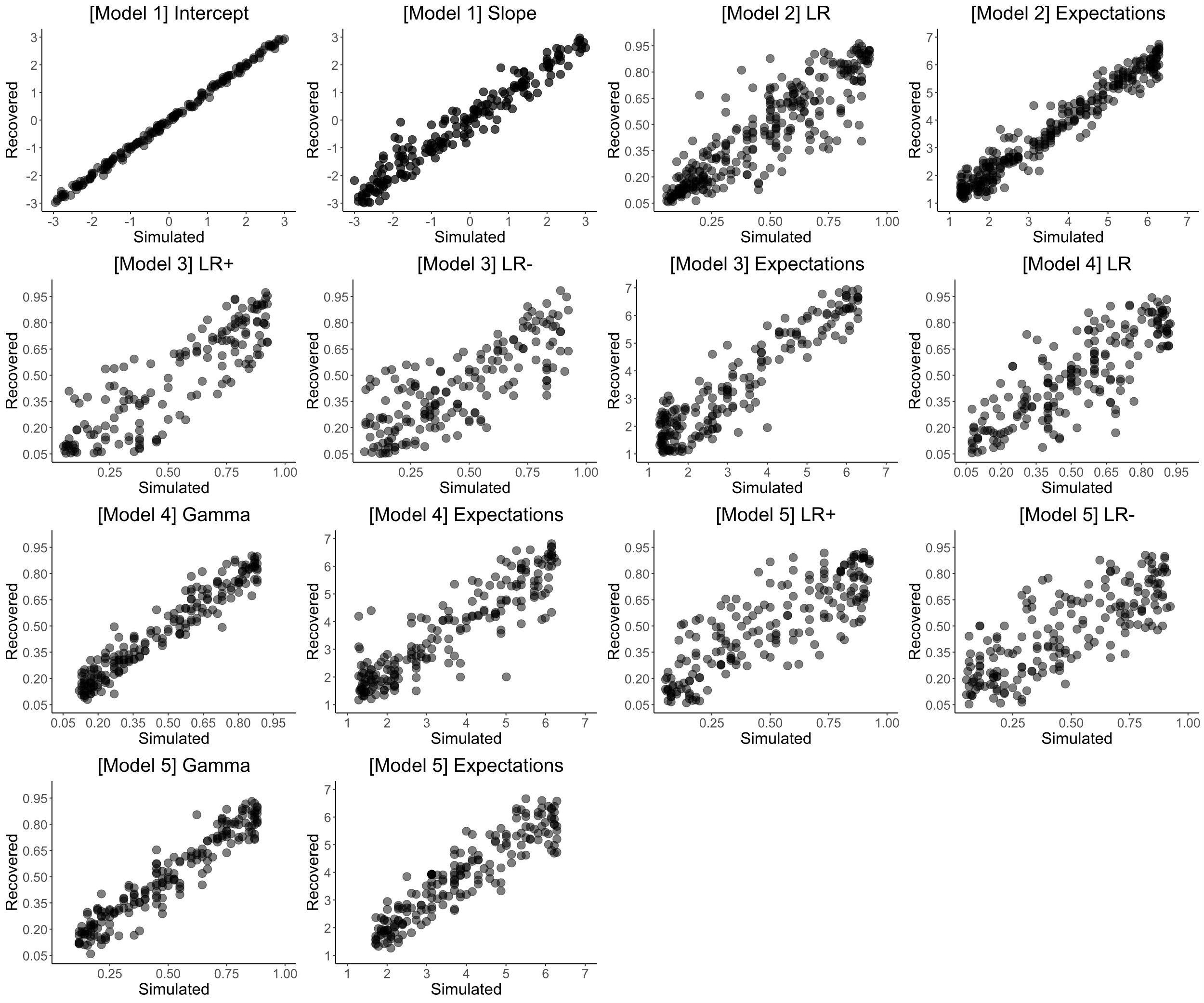** |
| --- |
| **Figure S2. Parameter Recovery.** Scatter plots representing the relationship between simulated (x-axis) and recovered (y-axis) parameters. |

| 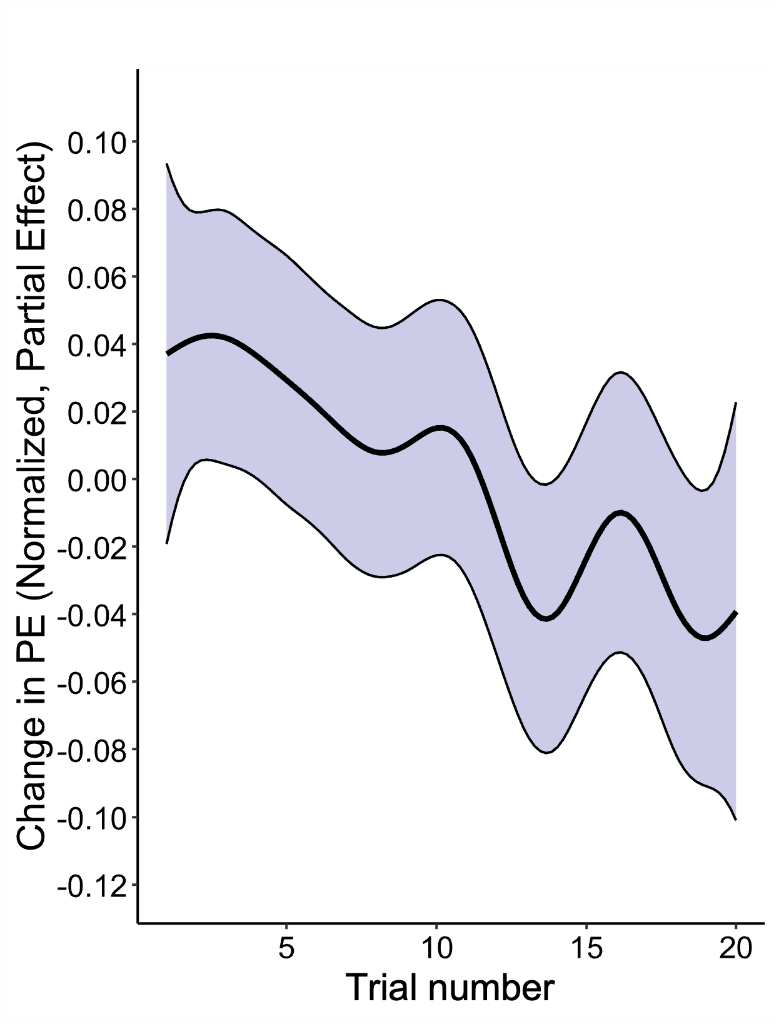 |
| --- |
| **Figure S3**. **Results from generalized additive model predicting prediction errors (PE) with trial number.** The effect of time was statistically significant (p < .05), indicating that participants learned during the task. Note that PEs themselves are absolute values, the y-axis represents the normalized difference according to the mean of the smooth term. |

| 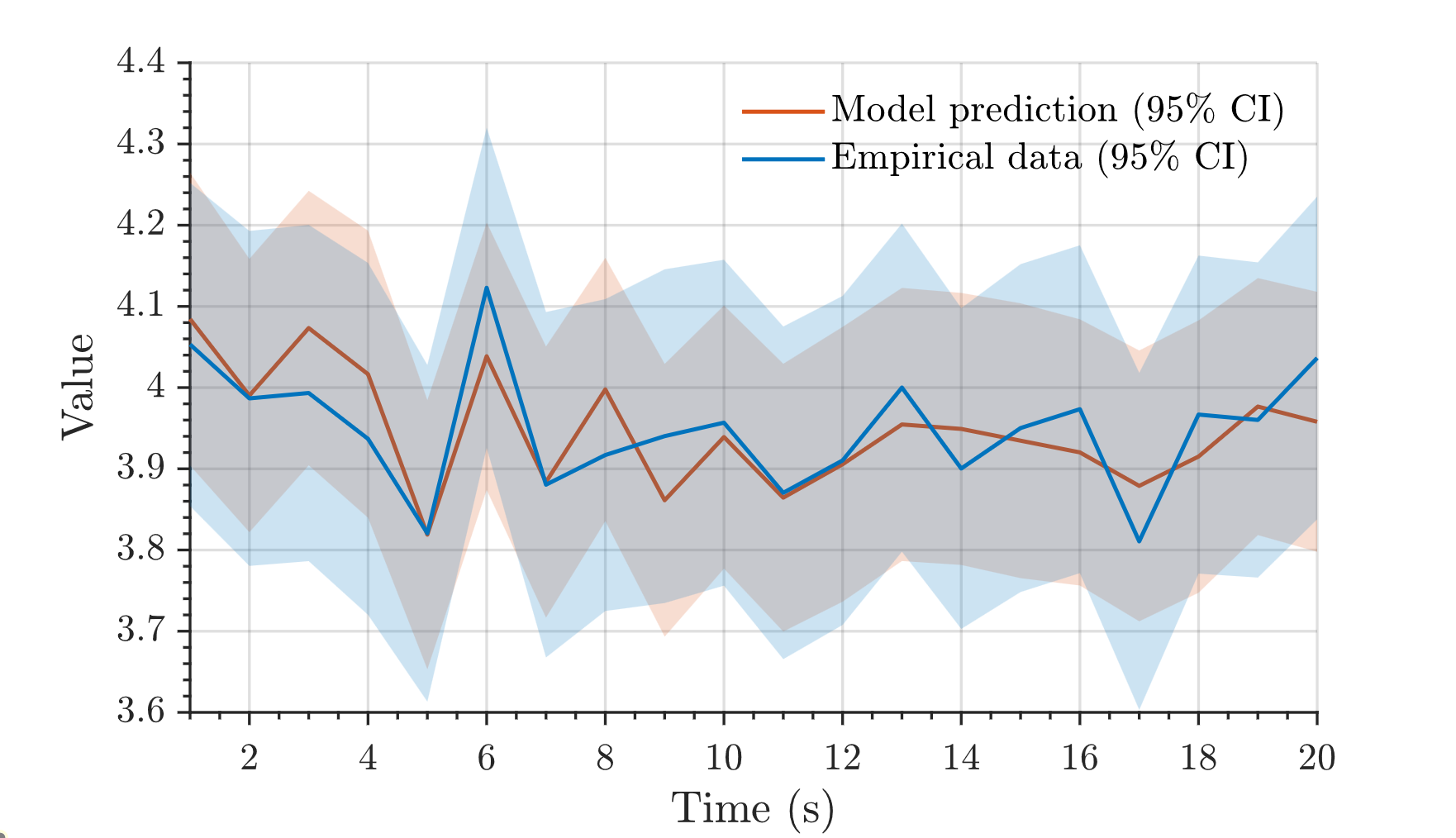 |
| --- |
| **Figure S4. posterior predictive checks for Model 4.** The orange solid line is the model’s posterior-predictive mean and the orange shaded band its 95 % confidence interval; the blue solid line is the observed (empirical) mean trajectory and the blue shaded band its 95 % CI. |

**Supplementary Note 1. Task Instructions**

The study was administered online and followed a fixed sequence of blocks:

1-Consent: Participants read an information sheet stating that participation was voluntary, data would be pseudonymous, and that they could withdraw at any time without penalty. Ethics approval from the University of Barcelona Research Ethics Committee was explicitly noted. Participants provided consent before proceeding.

2-Block 1: Initial ratings.

-Instructions: “In the first block, you will rate how much you AGREE with each statement. For every item, pick a number from 1 to 7. 1 means Strongly DISAGREE 7 means Strongly AGREE”. Participants then rated a set of 20 pseudoscientific statements (e.g., “The application of magnetic fields on the body can be used to treat physical and emotional alterations”).

Illustrative example:


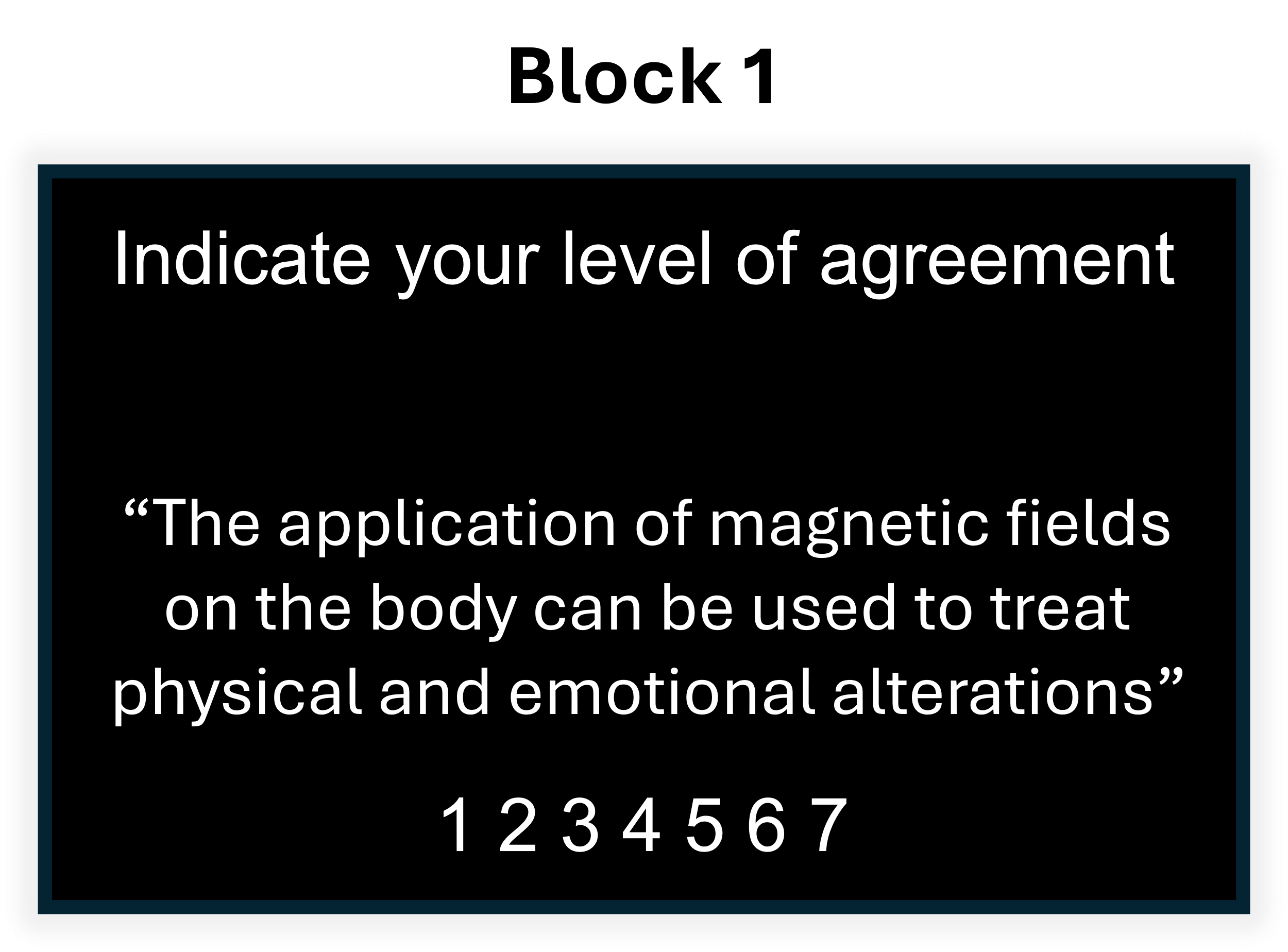


3-Distractor task: Participants solved a short series of simple arithmetic problems to provide a cognitive break and reduce immediate recall of earlier responses.

4-Block 2: Ratings with normative feedback. Instructions: “Your task here is the same as in Block 1. However, after you record each rating, wait a few seconds, and a screen will appear showing how strongly other people from a US representative sample agreed with that statement (mean agreement in a scale from 1 to 7)”. Participants again rated the same 20 items, with feedback appearing after each response. Illustrative example:


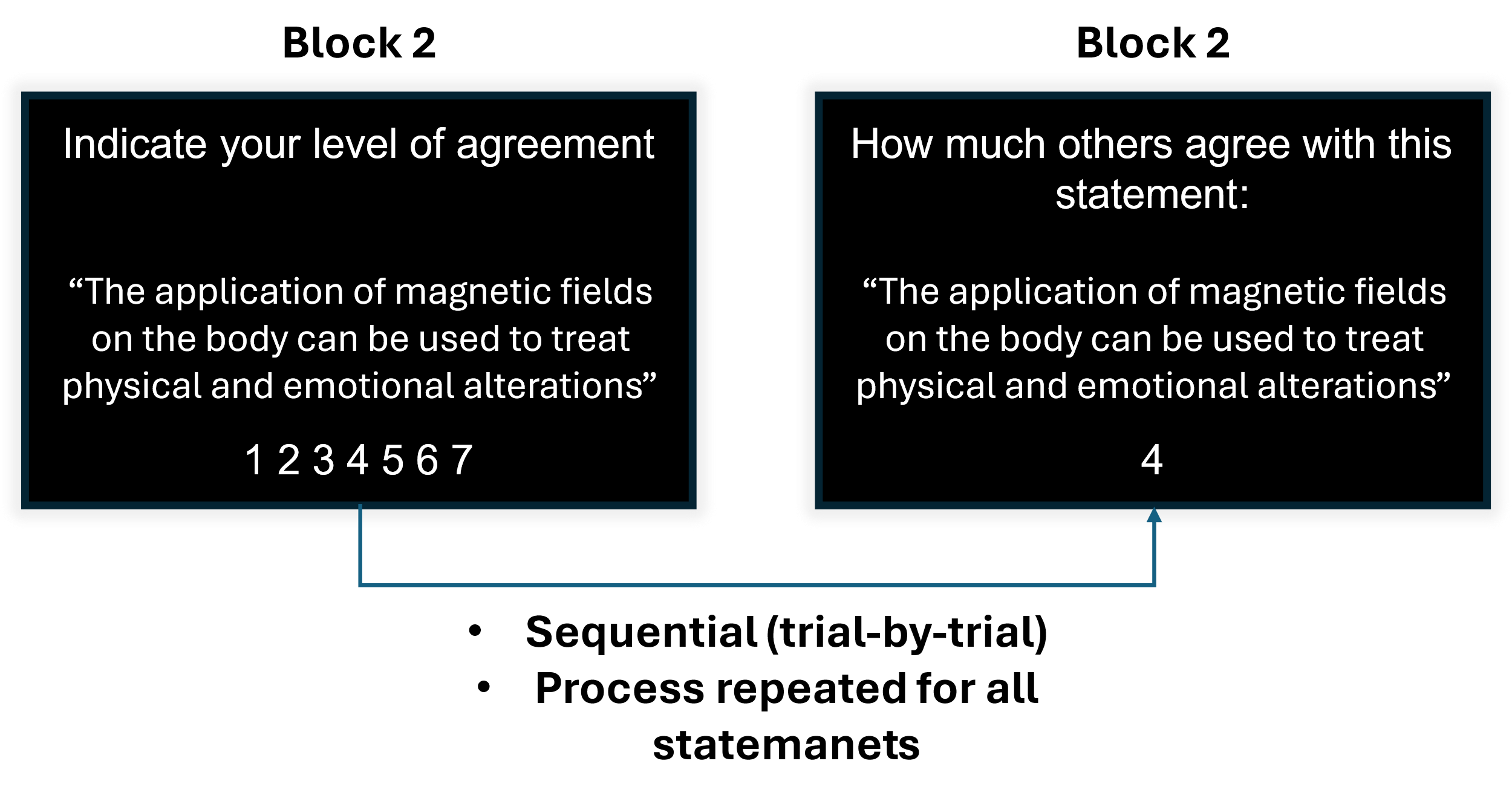


5-Debriefing and completion: A final screen thanked participants and explained that the study investigated how exposure to others’ views may influence personal beliefs. Participants were then redirected to Prolific for completion confirmation.
